# Xrn1-resistant RNA motifs are disseminated throughout the RNA virome and are able to block scanning ribosomes

**DOI:** 10.1038/s41598-023-43001-4

**Published:** 2023-09-25

**Authors:** Ivar W. Dilweg, Jasper Peer, René C. L. Olsthoorn

**Affiliations:** Leiden Institute of Chemistry, Leiden University, Einsteinweg 55, 2333CC Leiden, The Netherlands

**Keywords:** Ribosome, RNA

## Abstract

RNAs that are able to prevent degradation by the 5’–3’ exoribonuclease Xrn1 have emerged as crucial structures during infection by an increasing number of RNA viruses. Several plant viruses employ the so-called coremin motif, an Xrn1-resistant RNA that is usually located in 3’ untranslated regions. Investigation of its structural and sequence requirements has led to its identification in plant virus families beyond those in which the coremin motif was initially discovered. In this study, we identified coremin-like motifs that deviate from the original in the number of nucleotides present in the loop region of the 5’ proximal hairpin. They are present in a number of viral families that previously did not have an Xrn1-resistant RNA identified yet, including the double-stranded RNA virus families Hypoviridae and Chrysoviridae. Through systematic mutational analysis, we demonstrated that a coremin motif carrying a 6-nucleotide loop in the 5’ proximal hairpin generally requires a YGNNAD consensus for stalling Xrn1, similar to the previously determined YGAD consensus required for Xrn1 resistance of the original coremin motif. Furthermore, we determined the minimal requirements for the 3’ proximal hairpin. Since some putative coremin motifs were found in intergenic regions or coding sequences, we demonstrated their capacity for inhibiting translation through an in vitro ribosomal scanning inhibition assay. Consequently, this study provides a further expansion on the number of viral families with known Xrn1-resistant elements, while adding a novel, potentially regulatory function for this structure.

## Introduction

RNA viruses make use of a large variety of structures that may influence, or even hijack their host’s cellular mechanisms, ranging from transcription to translation and antiviral immune responses^1–6^. A relatively novel type of viral RNA structure has been discovered in the 3’ untranslated regions of an expanding number of flaviviruses and plant viruses^7–10^. These Xrn1-resistant RNAs (xrRNAs) have the capacity of resisting the highly processive RNA degradation of 5’ to 3’ exoribonuclease Xrn1^11^. Through this resistance, genomic RNA further downstream is protected, resulting in the accumulation of subgenomic RNA (sgRNA) species^7,12,13^. A distinct type of xrRNA termed ‘coremin’ (xrRNA_C_) was discovered in Beet necrotic yellow vein virus (BNYVV)^14,15^. This conserved RNA motif was determined to be necessary for accumulation of the sgRNA ncRNA3, through the action of stalling degradation by Xrn1 from the 5’ side. In BNYVV, this ability of xrRNA_C_ to stall Xrn1 influences viral RNA silencing suppression mechanisms, and is essential for the virus to achieve long-distance movement through the plant^16^.

Contrary to the large structures that hold an analogous function within the Flaviviridae^17,18^, and the Tombusviridae and Solemoviridae (xrRNA_LT_)^19,20^, no structure of xrRNA_C_ has been solved yet. Initial analysis of xrRNA_C_ motifs within Beny- and Cucumoviruses revealed a conserved sequence of a 4-bp hairpin (hp1) carrying a 4-nt loop (lp1), followed by a spacer of 8–10 nucleotides^21^. Subsequent work focusing on the BNYVV RNA3 xrRNA (xrRNA_BNYVV_) has elucidated more specifically its structural and sequence-specific requirements for stalling Xrn1^10^. It was determined that a second hairpin (hp2) which holds only structural conservation has to follow the conserved hp1 and spacer. Furthermore, Xrn1 resistance was retained only when lp1 followed the consensus YGAD, showing that some variation is possible within xrRNA_C_ that was not reflected in the sequences found in nature so far. These parameters lead to the discovery of novel xrRNA sequences within a variety of plant virus families^10^. With more xrRNAs being identified in an expanding number of viruses, we wondered to what extent certain variations of this motif are distributed throughout positive-sense, single-stranded RNA viruses, or even beyond. In this study, we show that xrRNA_C_-like motifs are present in untranslated regions and also intergenic regions (IGRs) of the genomes of several distantly-related viruses. Several of these novel putative xrRNA sites represent the first case of such sequences in double-stranded RNA (dsRNA) viruses. Notably, these candidate xrRNAs carried five or more nucleotides as lp1, diverging from the canonical four found in xrRNA_BNYVV_. Through in vitro degradation assays within either the xrRNA_BNYVV_, or the original genomic context, we show how such lp1 identities influence the Xrn1 resistance of xrRNA_C_ structures. These experiments were coupled with an investigation into the importance of xrRNA_C_ hp2 in order to find the minimal size and stability required for retaining Xrn1 resistance.

Recent studies have emphasized how the presence of xrRNA_LT_ species in IGRs may lead to them ending up in the 5’ UTR of sgRNAs, either due to synthesis from subgenomic promoters, transcription from prematurely terminated negative-strand RNA, or from incomplete degradation by Xrn1^22^. Consequently, the Tombusviridae that house such IGR xrRNA_LT_s may make use of their 3’ cap-independent translation enhancer elements in order to produce translationally active sgRNAs that are protected by xrRNAs^23–25^. Furthermore, the increasingly widespread distribution of xrRNA species and the short sequence required for its function in the case of xrRNA_C_, highlights how such structures may play a regulatory role in any RNA, coding or non-coding. These factors prompted us to investigate to what extent these structures are able to stall scanning ribosomes, and how this compares to stalling of Xrn1. We show how xrRNA_C_ indeed stalls scanning ribosomes, and that this ability is lost when mutations known to prevent stalling of Xrn1 are introduced. However, a true correlation between Xrn1 resistance and ribosomal stalling could not be demonstrated, since several xrRNA_C_ variants that have been shown to stall Xrn1, actually lost some ribosomal stalling ability. Overall, this study helps to further map the consensus of a functional xrRNA_C_, which should support the identification of novel instances of such sequences. The ability of these sequences to stall scanning ribosomes augments our understanding of the stalling mechanism by xrRNA_C_ and provides an explanation for the genomic location in which these structures may be found.

## Materials and methods

### BLAST searches

BLAST searches^26^ were carried out using variants of the original coremin motif^10^ GUCCGAAGACGUUAAACUAC. In these variants the 2nd and 3rd base pairs from the top were individually replaced by other Watson–Crick base pairs, or the loop size was extended by inserting N, NN, NNN, or NNNN at various positions in the YGAD consensus. These variants were used to screen several families of RNA viruses, adjusting the scoring parameters for mismatches and gaps. Hits were manually checked for strand polarity, location in the genome and presence of a putative hp2.

### Design and production of DNA templates and in vitro RNA transcription

Templates for production of RNA to be tested for Xrn1 resistance were designed and produced as described in Dilweg et al.^10^, using forward and reverse oligos that were purchased from SigmaAldrich in desalted form, with reverse complementary 3’ ends that allow compatibility in PCR reactions (see Supplemental data). This method yields products with a T7 promoter sequence (GTAATACGACTCACTATA), followed by a 12 nt leader sequence and the construct of interest, as depicted in Fig. 2. Production of DNA templates was validated by agarose gel electrophoresis and subsequently purified by ethanol/NaAc precipitation. Templates for CGAAAU, CGAAAC, CGAAAG, ACGAA and CGAAAAA lp1 constructs (Fig. 3) were instead acquired from the pMRL-derived plasmids that were produced for in vitro ribosomal scanning inhibition assays (Fig. 5). These carried a NcoI restriction site just downstream of the construct inserts. Thus, after linearization with NcoI, the product carried a T7 promoter sequence, followed by a 21 nt leader sequence (GGCTAGTTAAGATATAACATT) and the construct of interest. About 100 ng of linearized plasmid was used for run-off transcription. In vitro transcription was carried out for 30 min at 37 °C using T7 RiboMAX™ Large Scale RNA production System (Promega) for both template types. Reaction mixtures were treated with 1 unit RQ1 RNase-free DNase for 20 min at 37 °C. Transcript concentration was checked on agarose gel.

### In vitro Xrn1 degradation assay

Per reaction, about 200 ng of transcript was treated either with or without RppH and Xrn1 (both New England Biolabs), as described earlier^10^. After the addition of an equal volume of denaturing loading buffer (8 M urea, 20 mM Tris–HCl, 20 mM EDTA, trace amounts of bromophenol blue and xylene cyanol FF), RNA was denatured for 5 min at 75 °C. These samples were run on 8 M urea 14% polyacrylamide gels in TBE buffer, equilibrated at 60–65 °C. In the case of MRV JP-B and PicaV-C constructs (Fig. 2B), 14% non-denaturing polyacrylamide gels in TAE buffer were used instead, as described in Dilweg et al.^10^. Gels were stained with EtBr and most constructs were subjected to this assay at least twice. Band intensities were quantified using ImageJ software.

### Design of pScan and production of *Renilla* luciferase mRNA

The *Renilla* luciferase reporter vector pMRL^27^ was digested with HindIII and MfeI in order to insert pairs of complementary oligonucleotides that introduced KspAI and Van91I restriction sites between the T7 promoter sequence and the start codon of the *Renilla* luciferase ORF. Digestion of the resulting plasmid with KspAI and Van91I allowed insertion of pairs of complementary oligonucleotides that housed the xrRNA_BNYVV_-derived constructs of interest. Subsequent linearization on the XhoI-site downstream of the *Renilla* luciferase ORF, and purification with the PureYield™ Plasmid Miniprep System (Promega), yielded products suitable for run-off transcription as described above, though at minimum 1 h incubation time was applied in order to allow for production of the larger transcript. Reaction mixtures were treated with 1 unit RQ1 RNase-free DNase for 20 min at 37 °C, after which 20 μL Milli-Q water was added. Free nucleotides were removed by filtration through illustra™ MicroSpinTM G-25 columns (GE Healthcare), and RNA concentration of the flowthrough was determined by measuring absorbance at 260 nm and checking by agarose gel electrophoresis. Transcript concentration was diluted to 25 ng/μL with Milli-Q water.

### In vitro ribosomal scanning inhibition assay

For each measurement, a premix was prepared containing per sample 5 μL nuclease-treated rabbit reticulocyte lysate (Promega), 0.5 μL of 1 mM amino acid mixture without methionine, 0.5 μL of 1 mM amino acid mixture without lysine and 2 μL of 30-fold diluted Renilla-Glo™ Luciferase Assay Substrate (coelenterazine, Promega). Of this mixture, 8 μL was transferred to a 96-wells reaction plate per construct, in triplicate. To each well, 2 μL of 25 ng/μL RNA was added and mixed well, with intervals of 10 s. Luminescence was measured continuously for at minimum 100 min on a GloMax® Microplate Reader, with appropriate intervals between each well. For each time-point, means were calculated and normalized against the maximum mean luminescence reached by the sp.mut construct (Fig. 5) in order to gain values for relative luminescence, and to correct for differences in absolute luminescence between experiments.

## Results

### BLAST-searches for novel xrRNA_C_ motifs

**Figure 1:**
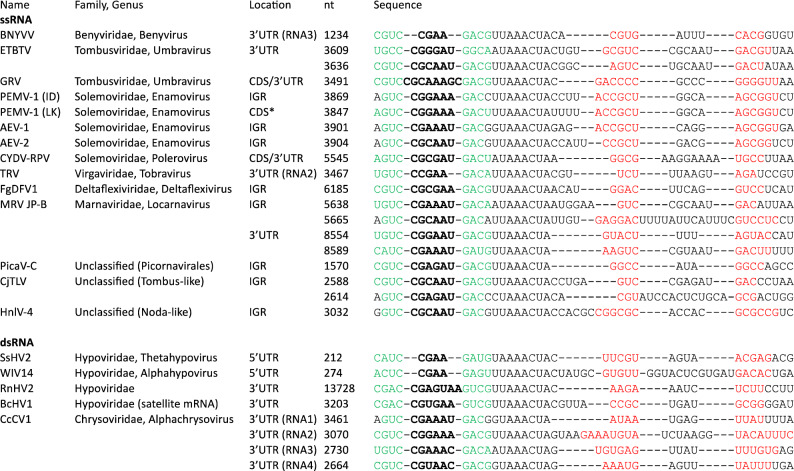
Overview of novel putative xrRNA_C_ motifs. Hp1 stem and loop are indicated in green and bold black font respectively, hp2 in red font. *IGR* intergenic region, *CDS* coding sequence. Asterisk: the motif is present in an automatically annotated hypothetical 34 codons ORF in the 3’UTR of sgRNA1. The number in column ‘nt’ corresponds to the first nucleotide of the sequence shown. Repeats of xrRNA_C_ motifs are divided over separate lines, each starting at a potential hp1. *BNYVV* beet necrotic yellow vein virus (KX665538), *ETBTV* Ethiopian tobacco bushy top umbravirus isolate 18-2 (KJ918748), *GRV* groundnut rosette umbravirus (MG646923), *PEMV-1* pea enation mosaic virus-1 (ID strain: HM439775, *LK* (Landkreis Meissen) strain: MN497826), *AEV-2* alfalfa enamovirus 2 (KY985463), *AEV-1* alfalfa enamovirus 1 (KU297983), *CYDV-RPV* cereal yellow dwarf virus-RPV (EF521830), *TRV* tobacco rattle virus (Z36974), *FgDFV1* *Fusarium graminearum* deltaflexivirus 1 (KX015962),* MRV JP-B* marine RNA virus JP-B (EF198242), *PicaV-C* picalivirus C (JQ898336), *CjTLV* Changjiang tombus-like virus 3 (KX883095), *HnlV-4* Hubei noda-like virus 4 (KX883214), *SsHV2* *Sclerotinia sclerotiorum* hypovirus 2 (MH347276), *WIV14* Wuhan insect virus 14 (KX883007), *RnHV2* *Rosellinia necatrix* hypovirus 2 (LC333745), *BcHV1* *Botrytis cinerea* hypovirus 1 (MG554634), *CcCV1* *Chrysothrix chrysovirus* 1 (RNA1: MN625832, RNA2: NC_055656, RNA3: MN625834, RNA4: MN625835).

In an attempt to expand on the currently known set of Xrn1-resistant RNAs, we used the previously determined structural requirements and sequence consensus of xrRNA_BNYVV_ for GenBank BLAST- searches against all RNA viruses. These searches focused on the more conserved hp1 and spacer portion of this motif, and hits were subsequently reviewed manually by assessing the genomic location (e.g. intergenic region (IGR) or untranslated region (UTR)) and the presence of a second hairpin following directly downstream of the conserved spacer sequence (Fig. 1). Of note, several candidate sequences carried, besides the regular motif characteristics, an lp1 consisting of five, six, seven or eight nucleotides instead of the four found in xrRNA_BNYVV_.

These hits provide for several viral families a first indication for the presence of an xrRNA within their genome. Notably, the double-stranded RNA (dsRNA) virus families of Hypoviridae and Chrysoviridae carry several candidate xrRNA sequences, within the 3’ UTR of Rosellinia necatrix hypovirus 2 (RnHV2), in a satellite-like RNA derived from Botrytis cinerea hypovirus 1 (BcHV1), and in the 3’ UTRs of all four Chrysothrix chrysovirus 1 (CcCV1) genomic RNAs. For the hits found within Tombusviridae and Solemoviridae, this represents the first time that two different types of xrRNA are found within a viral family, as both families house xrRNA_LT_-type structures as well^22^. Intriguingly, the Polerovirus CYDV-RPV appears to house both an xrRNA_LT_ in its IGR and a putative xrRNA_C_ around the stop codon of its most downstream ORF. While xrRNA_C_ was only identified in 3’ UTRs before, here we identified multiple hits in IGRs and even a few in coding sequences (CDS). Of note is Pea enation mosaic virus-1 (PEMV-1), which carries a putative xrRNA_C_ in its IGR for the isolate ID, or in a 3’UTR in isolate LK.

### Xrn1 resistance of novel, putative xrRNA_C_ hits

**Figure 2:**
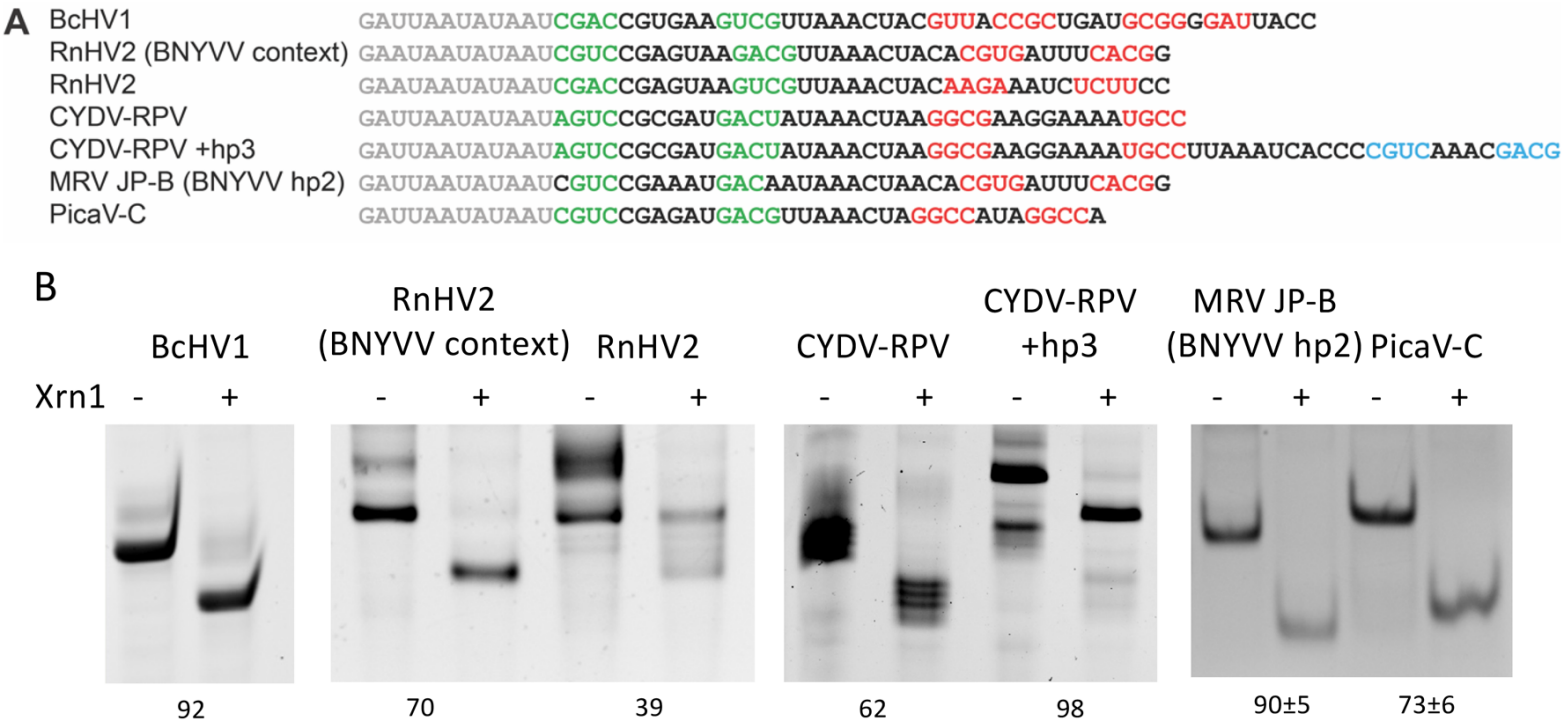
(**A**) Sequences of constructs that were tested for in vitro Xrn1 degradation assays based on the BLAST results listed in Fig. 1. The 5’ leader sequence is given in grey, and the predicted hp1 and hp2 stems in green and red, respectively. An additional hairpin identified downstream of CYDV-RPV xrRNA is given in blue. (**B**) Denaturing (or non-denaturing, in the case of the fourth gel from the left) polyacrylamide gels showing the results for in vitro Xrn1 degradation on the constructs listed in (**A**). Data below the gels indicate the average (± SD) percentage of Xrn1-resistant RNA. BcHV1, RnHV2 and CYDV-RPV constructs were measured only once.

To verify whether these novel xrRNA_C_ -like motifs function as actual xrRNAs, we tested Xrn1 resistance for some of the hits within their original contexts, using the exact hp1, spacer and hp2 sequence as they occur in the genome (Fig. 2A). Although in plants Xrn4 is the major exoribonuclease we used yeast Xrn1 as this enzyme has been found to resemble Xrn4 in many aspects^19,28^. Resistant constructs correspond with downshifted bands, where the single-stranded leader that is added to the construct is degraded, and Xrn1 is stalled at the nucleotide preceding hp1. All putative xrRNAs tested here appeared to show Xrn1 resistance (Fig. 2B). The heptaloop of RnHV2 appears Xrn1-resistant, although not all RNA has been subjected to degradation. This indicates that Xrn1 has not been able to associate with the construct, perhaps due to the A/U-rich nature of its hp2 forming interactions with the leader that prevent proper folding of the construct and Xrn1 landing on a single-stranded 5’ end. Replacing the unstable hp2 by that of BNYVV. However, in the context of xrRNABNYVV the CGAGUAA loop clearly confers Xrn1 resistance. We note that also MRV JP-B contains the BNYVV hp2 since we anticipated problems with its A/U-rich hp2 as well (Fig. 1). The CYDV-RPV construct also appeared to be Xrn1-resistant despite consisting of multiple bands, a problem that was likely caused by sequence specific 3′ terminal addition of nucleotides by T7 RNA polymerase during transcription. This was largely solved by extending the DNA template and hence the RNA with an additional native hairpin (CYDV-RPV + hp3).

### Interrogating the lp1-variation of xrRNA_C_

**Figure 3:**
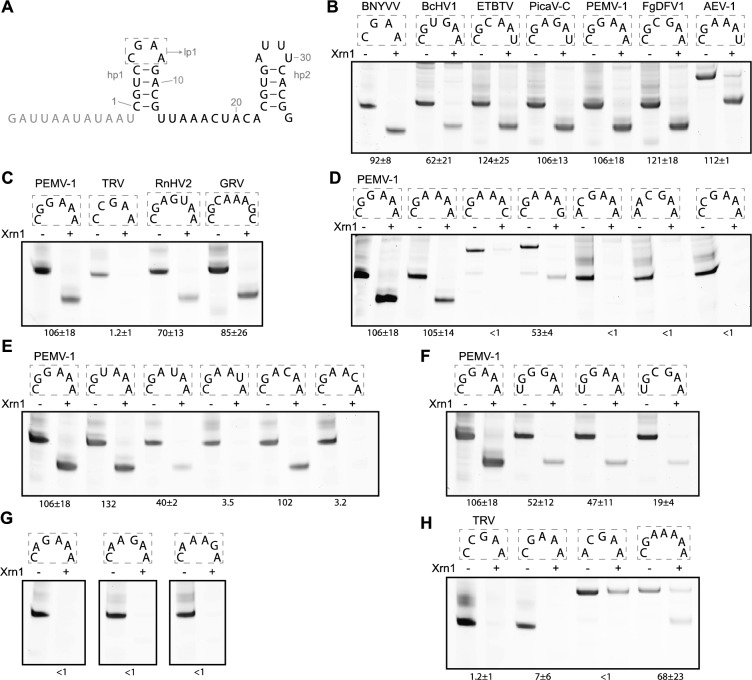
(**A**) Template construct used for in vitro Xrn1 degradation assays based on xrRNA_BNYVV_ with the predicted secondary structure arrangement illustrated and the 5’ leader sequence given in grey. (**B–H**) Denaturing polyacrylamide gels showing the results for in vitro Xrn1 degradation assays aimed at lp1 variants. Boxes above the gels depict what lp1 variants are tested in the corresponding lanes. RNA constructs are treated either with ( +) or without (−) RppH and Xrn1. Note that the CGAAAU, CGAAAC, CGAAAG, ACGAA and CGAAAAA lp1 constructs were derived from plasmids used for the in vitro translation assays (see “Materials and methods” section), and thus did not have the same initial length. Data below the gels indicate the average (± SD) percentage of Xrn1-resistant RNA. Certain constructs in (**E**) were measured only once.

The above results seemed to indicate that even in the context of BNYVV xrRNA a pentaloop confers functional Xrn1 resistance. To make a direct comparison of the effect of loop composition on Xrn1 resistance possible we tested several loop variants in the BNYVV context (Fig. 3A). The constructs carrying lp1 hexaloops CGUGAA (BcHV1), CGCAAU (ETBTV, AEV-2, CjTLV & HnlV-4), CGAGAU (PicaV-C), CGGAAA (PEMV-1), CGCGAA (FgDFV1) and CGAAAU (AEV- 1, MRV JP-B & RNA1 of CcCV1) were all clearly resistant to Xrn1 (Fig. 3B), as well as the heptaloop CGAGUAA (RnHV2) and the octaloop CGCAAAGC (GRV, Fig. 3C). In contrast, the pentaloop CCGAA (TRV), did not show Xrn1 resistance.

The strict requirements for specific nucleotides in the xrRNA_C_ hp1 and spacer sequence invited us to determine to what extent the hexaloop sequence could be varied without disturbing the Xrn1 resistance. Since the fourth nucleotide of the xrRNA_BNYVV_ tetraloop allowed for either an A, G or U^10^, and CGAAAU was resistant, we tested whether this applied to the hexaloop variant as well. Indeed, constructs carrying CGAAAA and CGAAAG were Xrn1-resistant, while CGAAAC was not (Fig. 3D). This suggests that the most downstream hexaloop nucleotide fulfills the same function as the most downstream tetraloop nucleotide. Furthermore, ACGAAA and AACGAA constructs were made, to directly assess whether the additional two nucleotides of the hexaloop variant could be placed at any position without disturbing proper folding of the structure, and thus whether the CGAA motif of the tetraloop could just be shifted downstream. This does not seem to be the case, as these constructs did not retain Xrn1 resistance (Fig. 3D), indicating as well that the most upstream loop nucleotide is involved in the same interaction within either the hexaloop or the tetraloop xrRNA_C_ variants. Interestingly, a CCGAAA loop, carrying the CGAA tetraloop motif combined with an additional C at the first hexaloop position, does not stall Xrn1 either (Fig. 3D), which either indicates that the G in the third hexaloop position cannot substitute for missing a G in the second position or that the C in the second position is disruptive for proper folding. Both arguments provide a potential explanation for why the pentaloop CCGAA did not show proper Xrn1 resistance either.

In order to further characterize the hexaloop variant and to compare it to the tetraloop variant, constructs containing CGUAAA, CGAUAA, CGAAUA were tested, since in the tetraloop xrRNA_C_ a CGUA lp1 was not Xrn1-resistant^10^. From these constructs, only CGAAUA did not retain Xrn1 resistance, suggesting that within the hexaloops, the fifth nucleotide fulfills the role of the third tetraloop nucleotide (Fig. 2E). This hypothesis was tested further by assessing the Xrn1 resistance of constructs containing a CGACAA or CGAACA loop, corresponding with the non-functional CGAC tetraloop lp1. Indeed, the fact that CGACAA retains Xrn1 resistance, while CGAACA and CGAAAC do not (Fig. 3E), appears to confirm this hypothesis, while also indicating that hexaloop position 4 could be any nucleotide, especially considering the tested natural hexaloops appear to follow this trend as well. While the natural hexaloop sequences discovered all contained a C as their first loop nucleotide, the tetraloop xrRNA_C_ was previously determined to allow for a YGAD consensus. The variants UGGGAA, UGGAAA and UGCGAA were tested in order to see if this were true for the hexaloop xrRNA_C_ constructs as well (Fig. 3F). When compared to their C-carrying counterparts, this was the case, although the amount of RNA leftover did decrease significantly, comparable to what occurs for a UGAA lp1^10^. The partial resistance of UGGGAA, UGGAAA, UGCGAA, and CGAAAG (Fig. 3D) is possibly due to the formation of an additional base pair between positions 1 and 6 in the loop, leading to a stable GNRA tetraloop that is interfering with the function of the hexaloop.

In comparing the tetraloop xrRNA_C_ YGAD consensus with a hexaloop counterpart, a next set of constructs was aimed at figuring out whether the necessary position of the tetraloop GA is allowed to be changed within the hexaloop equivalent. As such, CAGAAA, CAAGAA and CAAAGA loops were tested, and all turned out to be unable to stall Xrn1 (Fig. 3G), indicating that the position of the loop G has to be retained. Previous work has indicated that within the tetraloop xrRNA_C_, a CAGA loop is not Xrn1-resistant either^10^.

Overall, the hexaloop variants tested in this study point towards a conservation of the tetraloop xrRNA_C_ YGAD consensus, with any two nucleotides in between the second and third positions (YGNNAD). Considering this knowledge, we were curious to see whether it would be possible to rescue the non-functional CCGAA by testing a sequence that would follow YGNAD. This was not the case however, as CGAAA did not result in any Xrn1-resistant RNA (Fig. 3H). As expected from the non-functional ACGAAA hexaloop, an ACGAA was not Xrn1- resistant either. Furthermore, the Xrn1-resistant heptaloop variant of RnHV2 inspired us to test the more simple CGAAAAA loop, which did appear to be completely resistant (Fig. 3H), further indicating that the YGAD motif is necessary and could be extended through extra nucleotides in the middle, but that more than one extra nt is needed. We note that not all input RNA of ACGAA and CGAAAAA was digested by Xrn1; this is probably due to the use of a different leader sequence in these constructs (see also Materials and methods).

### Finding the minimal hp2 for xrRNA_C_

**Figure 4:**
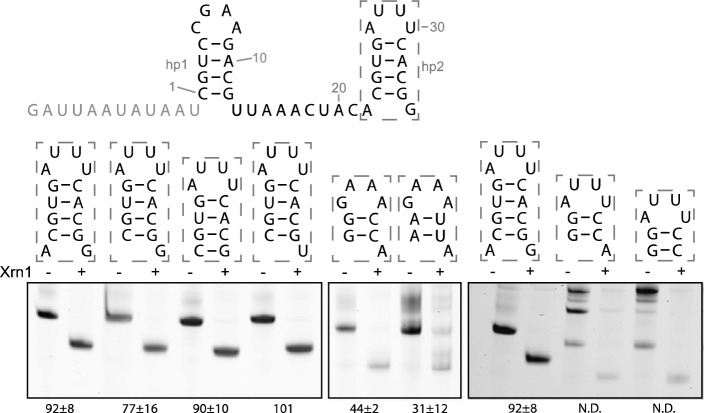
Template construct used for in vitro Xrn1 degradation assays based on xrRNA_BNYVV_ with the predicted secondary structure arrangement illustrated and the 5’ leader sequence given in grey is depicted above. Underneath are denaturing polyacrylamide gels showing the results for in vitro Xrn1 degradation assays aimed at hp2 variants. Boxes above the gels depict what hp2 variants are tested in the corresponding lanes. RNA constructs are treated either with ( +) or without (−) RppH and Xrn1. Data below the gels indicate the average (± SD) percentage of Xrn1-resistant RNA. N.D. indicates that the percentage could not be reliably determined.

Previous work on the xrRNA_C_ motif has established that the second hairpin hp2 is absolutely required for Xrn1 resistance, although it is not conserved at sequence level^10^. Finding novel putative xrRNA hits with comparable hp1 and spacer, but quite variable downstream sequences (Fig. 1) underscores the need for determining more exactly what is minimally required downstream to keep Xrn1 resistance. Overall, through systematic substitution of several elements within, or flanking hp2, we noticed that most changes allowed for sustained Xrn1 resistance (Fig. 4). These changes included deletion of the A22 flanking hp2, or both nucleotides flanking hp2; substituting G35 for a U; reducing hp2 down to two G-C bps, with either a thermodynamically stable GAAA, or a regular AUUU tetraloop. Although the latter construct still retained Xrn1 resistance, this variant showed many additional bands indicating either undenatured or misfolded intermediates, making the analysis less reliable. Likewise the removal of the 3’A from the latter construct (Fig. 4, last two lanes) or replacing the two G-C bps by A-U bps still allowed for Xrn1 resistance but also led to the appearance of additional bands that were not Xrn1 resistant. From this we conclude that a 2-bps hp2 is sufficient to stall Xrn1 but it should preferably by capped by a stable tetraloop.

### Stalling scanning ribosomes by xrRNA_C_

**Figure 5:**
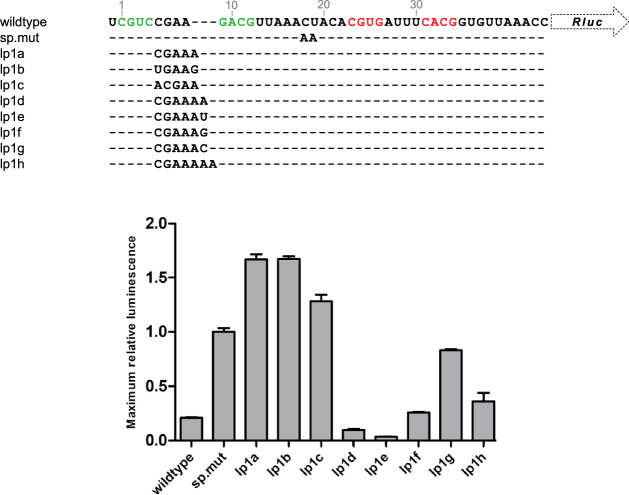
In vitro ribosomal scanning inhibition assay. Mutations relative to the wildtype are given underneath, with dashes indicating no change. The wildtype sequence is numbered in grey and shows nucleotides involved in stems of hp1 and hp2 in green and red, respectively. Maximum relative luminescence. Data are presented as mean of measurements in triplicate, normalized to the maximum luminescence reached by sp.mut, with error bars depicting ± SD.

Finding putative xrRNAs in IGRs and CDSs, and the ability to stall and prevent the helicase activity of Xrn1, led us to investigate whether xrRNA_C_ structures are able to stall scanning ribosomes. This was tested by cloning xrRNA_BNYVV_ and mutated versions within the 5’ UTR of a luciferase reporter plasmid (Fig. 5A). The mRNA derived from these plasmids was used in a rabbit reticulocyte lysate in vitro translation system, in which conversion of substrate by produced luciferase enzymes was tracked over time by detecting the luminescence resulting from this reaction. The degree of ribosomal stalling by xrRNA_BNYVV_ (wildtype) was determined through comparison with a construct carrying a substitution of the spacer C and U with two A’s (sp.mut), a version that has previously been shown to be unable to stall Xrn1^10^. It appears that the production of luciferase occurs much more rapidly within the sp.mut construct, leading to roughly a five-fold increase in maximum relative luminescence (MRL) after about an hour of translation compared to the wildtype construct (Fig. 5; see Supplementary figure S1 for time traces). This indicates that the identity of the spacer sequence also plays a crucial role in the extent to which ribosomal scanning can be delayed.

The Xrn1-digestion assays using xrRNA_C_ motifs with loops carrying five, six or seven nucleotides yielded the notion that pentaloops are mostly unable to stall Xrn1, whereas hexaloops could, given that they follow a YGNNAD consensus. The consensus for a heptaloop was not investigated as thoroughly, but both a CGAGUAA and a CGAAAAA loop were found to be Xrn1 resistant. Tracking the luminescence for the constructs carrying the pentaloops CGAAA (lp1a), UGAAG (lp1b) and ACGAA (lp1c), all show a strong loss of ribosomal stalling (Fig. 5), even worse than demonstrated by sp.mut. Conversely, hexaloop lp1 constructs revealed a measure of ribosomal stalling that roughly equals (lp1f, CGAAAG), or even surpasses (lp1d, CGAAAA; lp1e, CGAAAU) that of the wildtype construct. The CGAAAC lp1 (lp1g) instead reached a MRL of about 0.8 times that of sp.mut. These results suggest that the loss of Xrn1 resistance by several penta- and hexaloops, generally coincides with the loss of ribosomal stalling capacity, while the hexaloops found to be Xrn1 resistant show the potential of stalling ribosomes more than the wildtype xrRNA_BNYVV_ motif. A construct carrying the CGAAAAA heptaloop (lp1h) reached an MRL that was twice that of the WT construct, although still around 0.4 times the MRL of sp.mut, indicating that heptaloops in an xrRNA_C_ may partially retain the ability to stall ribosomal scanning.

Since many of these motifs were found in plant viruses, we investigated the main constructs also in a wheat germ extract. This led to basically the same results (Supplementary Fig. 2), demonstrating that these Xrn1-resistant motifs can impede ribosomal scanning irrespective of ribosome origin.

## Discussion

The relatively small size of the Xrn1-resistant coremin motif and the lack of structural information currently available, keeps how exactly Xrn1 is unable to progress through xrRNA_C_ elusive. The types of xrRNA found in Flaviviridae and Tombusviridae, have been characterized through mechanistic studies and crystal structures, indicating elaborate assemblies of stem-loops, pseudoknots, and additional tertiary interactions^17–19,28,29^ forming a ring-like structure that, when approached from the 5’ side, serves as a mechanical and topological blockade that Xrn1 cannot progress through. The initially predicted secondary structure configuration for xrRNA_BNYVV_, with two small hairpins separated by a spacer, does not easily inspire a way for imagining a similar mode of stalling. In the absence of crystallographic data, we have further characterized this motif through Xrn1 degradation assays on a large variety of constructs based on the xrRNA_BNYVV_. These have expanded the currently known distribution of putative xrRNA_C_s, and further clarified what is minimally required at the sequence and nucleotide level for Xrn1 to be stalled.

### Loop size matters

Through GenBank BLAST searches, several xrRNA_C_-like viral sequences were found that carried a lp1 of five or more nucleotides, instead of the xrRNA_BNYVV_ tetraloop. Most of the constructs containing such loops were Xrn1-resistant, either within the xrRNA_BNYVV_ context (Fig. 3), or in their own (Fig. 2). Systematic variation of hexaloop-containing xrRNA_C_ motifs led us to propose a consensus sequence of YGNNAD for the lp1. A notable exception to this consensus discovered through our BLAST searches however, are two of the four putative xrRNAs demonstrated within the Alphachrysovirus CcCV1. Both carry a C at the last position, which highlights the importance of testing these sequences within their own genomic context, and therefore cannot be conclusively used for determining the Xrn1 resistance of a similar motif. The lp1 consensus suggests that the middle two nucleotides bulge out, while the flanking nucleotides may be involved in an interaction with the spacer as proposed earlier for the YGAD consensus within xrRNA_BNYVV_^10^. This raises the issue whether the two—seemingly uninvolved—nucleotides have a non-structural function, or whether they are actually redundant. The fact that even the predicted GRV octaloop yields an Xrn1-resistant structure within the xrRNA_BNYVV_ context, suggests that more redundant nucleotides are allowed within lp1. Interestingly, the GRV octaloop ends with a C as well, which suggests that such a loop does not follow a YGNNNNAD consensus. Therefore, in this octaloop variant, the putative interaction that involves the most downstream nucleotide may instead involve a more upstream one. The viral context of the GRV candidate xrRNA is notable, with the predicted hp1 stem and spacer mostly identical to xrRNA_BNYVV_, immediately followed by a stable hp2. However, this motif is followed by the stop codon of an annotated hypothetical protein, suggesting it is part of a coding sequence. Therefore, in this particular case, the presence of more than four nucleotides in the loop sequence could be explained by their role as specific codons in translation. The pentaloop-carrying xrRNA_C_-like motif found in an isolate of TRV RNA2, was determined to not yield an Xrn1-resistant structure. This contrasts the Xrn1-resistant motif that carried the regular CGAA lp1 found in the TRV RNA2 isolate tested in our earlier study^10^. Only one isolate with a pentaloop was discovered through our BLAST searches, contrasting the number of hexaloops-containing motifs found, which suggests that the pentaloop-containing TRV xrRNA_C_-like motif may have evolved to become non-functional, or perhaps holds another function entirely.

### Correlation between 5′UTR located xrRNA motifs and presence of an IRES

Several putative xrRNAC were identified in dsRNA viruses of the Hypoviridae and Chrysoviridae families (Figs. 1, 2B). To our knowledge, this shows for the first time that putative xrRNAs may exist outside the realm of single-stranded RNA viruses. The fact that all four genomic RNAs of CcCV1 carry an xrRNAC-like motif at the 5’ end of the 3’ UTRs is a strong indication for their Xrn1-resistant functionality. Due to only BLAST’ing against single-stranded RNA viruses in our previous study^10^, we now additionally identified tetraloop versions of the xrRNAC motif within the 5’ UTRs of Wuhan insect virus 14 and Sclerotinia sclerotiorum hypovirus 2, both members of the Hypoviridae family (Fig. 1). While the conservation of IRES-like elements in the 5’ UTR of Hypoviridae is yet uncertain, for several species within these mycoviruses IRES activity has been implied^30,31^, and the location of the xrRNA_C_ motifs within these species does allow for enough space between it and the polyprotein start codons. Therefore, the relationship between xrRNAs in 5’ UTRs and the presence of IRES structures reduces the chance for such motifs to encounter scanning ribosomes. However, translation initiation on multicistronic viral RNAs is not always accounted for, and it cannot be ruled out that undiscovered xrRNA_C_-like motifs, or other types of xrRNA, could serve a regulatory role within coding sequences of viruses or elsewhere. At the least, xrRNA_C_ being able to stall scanning ribosomes, provides an explanation for why most of these motifs presently known are located in the 3’ UTR, where they cannot interfere with translation processes for viruses that initiate from a 5’ cap. While most members of the Flaviviridae carry strongly conserved xrRNAs in their 3’ UTR^32,33^, xrRNAs have also been discovered in the 5’ UTR of Hepatitis C virus and Bovine diarrhea virus^34^. These viruses have an IRES downstream, which allow them to continue initiation of translation even after losing the 5’ cap and being subjected to Xrn1 degradation. Furthermore, this IRES allows them to bypass ribosomal scanning from the 5’ end, and thus would prevent the stalling that could occur from the xrRNA.

A recent study on the distribution of xrRNA_LT_s has demonstrated their presence throughout the Tombusviridae and Solemoviridae families^22^. Here we show how instead, at least two species of Umbravirus, ETBTV and GRV that belong to the Tombusviridae, carry a putative xrRNA_C_. As such, these findings indicate two divergent types of xrRNA present within the Umbravirus genus. Moreover, the Polerovirus CYDV-RPV carries both an xrRNA_LT_ in its IGR, and a putative xrRNA_C_ that is partly located within a CDS. Like the putative xrRNA of GRV, this latter motif embeds a stop codon Whether these sequences play a role in translation by slowing down of ribosomes thereby affecting nascent protein folding similar to e.g. G-quadruplexes^35^ or play a role in RNA silencing suppression, similar to xrRNA_BNYVV_, will require further study.

In this study, we show how xrRNA_BNYVV_ is able to stall scanning ribosomes, leading to a significantly lower production of luciferase compared to constructs that harbor mutations that are known to abolish their Xrn1 resistance (Fig. 5). Like xrRNA_LT_s discovered in earlier studies^22^, the putative xrRNA_C_ motifs found in this study are located both in IGRs and 3’ UTRs, and their function likely varies depending on this location. In order to regulate translation of their uncapped RNA, Tombusviridae make use of 3’ cap-independent translation enhancers^23,36,37^. This provides a potential role for xrRNAs located in IGRs, since subgenomic RNA that result from stalling of Xrn1 may retain a certain level of translational activity. As such, ORFs located on these subgenomic RNAs are subjected to translational regulation through either protection of the RNA from degradation, or—in the case of an xrRNA not located at the 5’ end of the subgenomic RNA—through stalling of the scanning ribosome. These findings, and the fact that novel xrRNA_C_ candidates are found within IGRs, and even (at the end of) CDSs, highlight the importance of mapping the interplay of translation regulation and Xrn1-mediated decay that viruses employ.

### Role of hp2

Most of the currently predicted xrRNA_C_ sequences allow for a relatively stable hp2 (Fig. 1). Exceptions are the second motif found in CjTLV (a 3-bp hp2 with a G-U loop-closing bp that is unlikely to form), and the A-U- and G-U-rich motifs of CcCV1. However, we have tested several hp2 mutants of the xrRNA_BNYVV_ in order to pinpoint what is minimally required for stalling Xrn1. This resulted in the conclusion that A22 and nucleotides downstream of hp2 were not essential for stalling Xrn1, and that a two-bp hp2 is sufficient provided it is capped with a stable tetraloop. Therefore, it can be deduced that the theoretical, shortest Xrn1-resistant sequence based on the xrRNA_C_ motif would be 5’-GUCCGAAGACGUUAAACUACGGGAAACCA-3’. It should be noted, however, that this would likely only be the case in vitro. Within the context of a highly structured UTR, this sequence would possibly not fold in such a way that the specific Xrn1-resistant topology could be stably maintained. So what exactly is the role of hp2 in stalling Xrn1? In flaviviral xrRNA, the pseudoknot involving its apical loop folds around the 5’ end from which Xrn1 approaches, causing the enzyme to brace against the ring-like topology, halting degradation^17,18,28^. If in the xrRNA_C_ motif, hp2 only functions to brace against the enzyme, it would explain why any small but stable hairpin retains the construct’s Xrn1 resistance. Xrn1 is halted at one nucleotide upstream of hp1^21^, which means that the first one or two nucleotides of hp1 actually enter the active site of the enzyme^38^. It is therefore unlikely that hp2 functions in a similar fashion, serving as a topological blockade for Xrn1, as the enzyme likely has to brace against the surface of hp1 in order reach the predicted stalling site. Conversely, the exclusively structural conservation, and this study showing the need for just a small hairpin, actually do suggest a mechanical function.

### Correlation between thermodynamic stability of xrRNA and stalling of ribosomes and Xrn1

Following the discovery that xrRNA_BNYVV_ is able to stall scanning ribosomes, we were eager to find out whether there is a positive correlation between that ability, and Xrn1 resistance. Most of the constructs tested for ribosomal stalling capacity indeed show that a loss of Xrn1 resistance also coincides with a loss of ribosomal stalling. This correlation is well pronounced for the substitution of spacer nucleotides 18 and 19 (sp.mut), which are known to be crucial for stalling Xrn1. Furthermore, the constructs testing the Xrn1-resistant hexaloop variants CGAAAA, CGAAAU and CGAAAG, indicated ribosomal stalling on par with, or better than the wildtype. In contrast, the non-Xrn1 resistant CGAAAC lp1 appears to lose this ability at least partially, reaching an MRL comparable to the spacer mutant. As such, these assays provide a potential for additional information on the structural integrity of xrRNA_C_ variants, where Xrn1-digestion assays do not account for structures ‘more’ resistant than wildtype. Furthermore, the pentaloop constructs, which are all unable to resist Xrn1, appear to slow down ribosomes even less than the spacer mutant, indicating an even stronger loss of thermodynamic stability and/or tertiary structure than caused by mutations in the spacer.

We must however take into account that certain substitutions within the xrRNA_BNYVV_ may influence not only thermodynamic stability of the construct, but also the ratio of functionally versus non- functionally folded structures. A recent study highlighted the importance of this folding process for Zika xrRNA, showing how misfolded intermediates without the intricate structure necessary for stalling Xrn1 may ultimately form^39^. Furthermore, Xrn1 and ribosomes do not process RNA in the same way. While Xrn1 is unable to progress through an xrRNA structure even after 20 h of incubation (Supplementary Fig. S3), this in vitro translation assay shows that in constructs that stall scanning ribosomes, luciferase is ultimately produced. This suggests that at least for a subset of mRNAs, the ribosomes are able to progress.

The manual assessment of novel xrRNA_C_ motifs from BLAST-searches is unlikely to be exhaustive, and it is therefore likely that more viruses carrying such structures can be found. Since the intrafamilial conservation of these motifs was not explored thoroughly, it remains unclear to what extent these structures are abundant throughout. The expansion of lp1 sequences that may confer Xrn1 resistance in the context of xrRNA_BNYVV_, and the minimal hairpin that is required, should aid further interrogation of viral genomes for these motifs. Knowing that they are able to stall scanning ribosomes, we may also look into the 5’ UTRs of viral families with internal translation initiation capacities. Conversely, how often xrRNA_C_ motifs are positioned in the genome such that they mostly evade ribosomes, as opposed to a position where they may provide a more regulatory, ribosome-inhibiting function, is yet open to question.

### Supplementary Information

Supplementary Figures.

## Data Availability

The authors confirm that the data supporting the findings of this study are available within the article and its supplementary materials.
