# Supplementary material for: Xrn1-resistant RNA motifs are disseminated throughout the RNA virome and are able to block scanning ribosomes
Source: Sci Rep. 2023 Sep 25;13:15987. doi: 10.1038/s41598-023-43001-4 (PMC10520033; doi:10.1038/s41598-023-43001-4)
Supplement: Supplementary file 1 — Supplementary Figures. [file 41598_2023_43001_MOESM1_ESM.docx]

**Supplemental data**

**Xrn1-resistant RNA motifs are disseminated throughout the RNA virome and are able to block scanning ribosomes**

*Ivar W. Dilweg, Jasper Peer, René C.L. Olsthoorn**

*Leiden Institute of Chemistry, Leiden University, Einsteinweg 55, 2333CC, Leiden, The Netherlands*

**corresponding author e-mail: olsthoor@chem.leidenuniv.nl*

**Supplementary Fig. S1.** In vitro ribosomal scanning inhibition assay. Mutations relative to the wildtype are given underneath. The wildtype sequence is numbered in grey and shows nucleotides involved in stems of hp1 and hp2 in green and red, respectively. Traces of measured luminescence over time. Data are presented as mean of measurements in triplicate, normalized to the maximum luminescence reached by sp.mut, with error bars depicting ± SD. The mean trace of the wildtype is shown in all graphs for comparison. Cubic spline curves were fitted to the data in order to guide the eye.


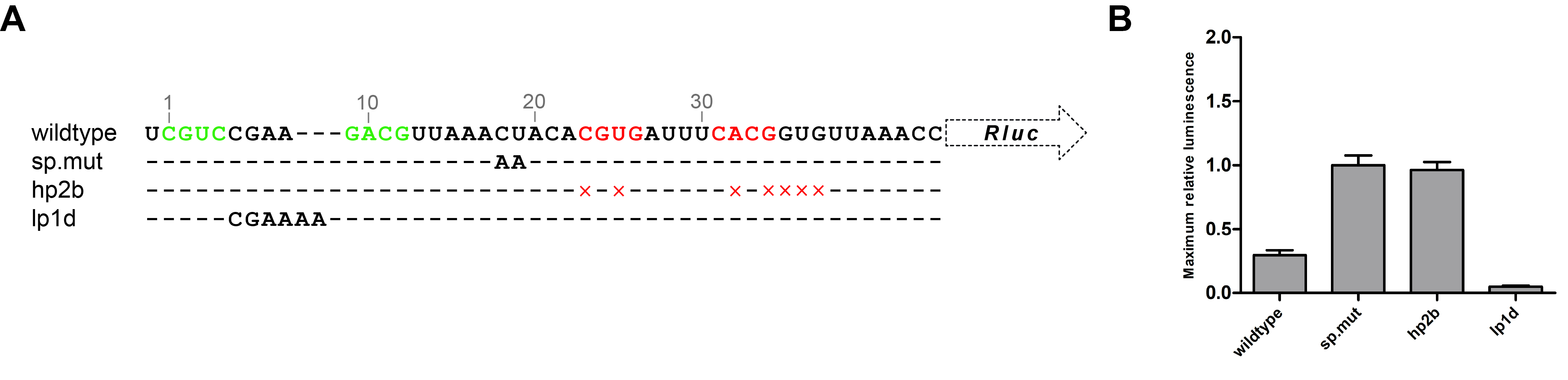
**Supplementary Fig. S2. (**A) Constructs tested in in vitro ribosomal scanning inhibition assay using wheat germ extract (Promega). Mutations relative to the wildtype are given underneath, with dashes indicating no change, and red crosses indicating deletions. The wildtype sequence is numbered in grey and shows nucleotides involved in stems of hp1 and hp2 in green and red, respectively. (B) Maximum relative luminescence. Data are presented as mean of measurements in triplicate, normalized to the maximum luminescence reached by sp.mut, with error bars depicting ± SD.


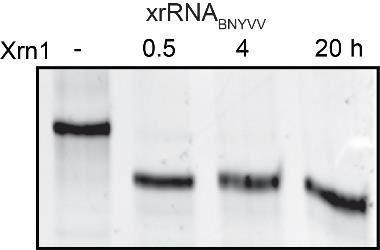


**Supplementary Fig. S3.** *In vitro* Xrn1 digestion assay on xrRNABNYVV preceded by a 12-nt leader sequence, with ~200 ng RNA incubated for increasing durations with RppH and Xrn1, as depicted above. Samples were loaded on a 14% denaturing polyacrylamide gel.


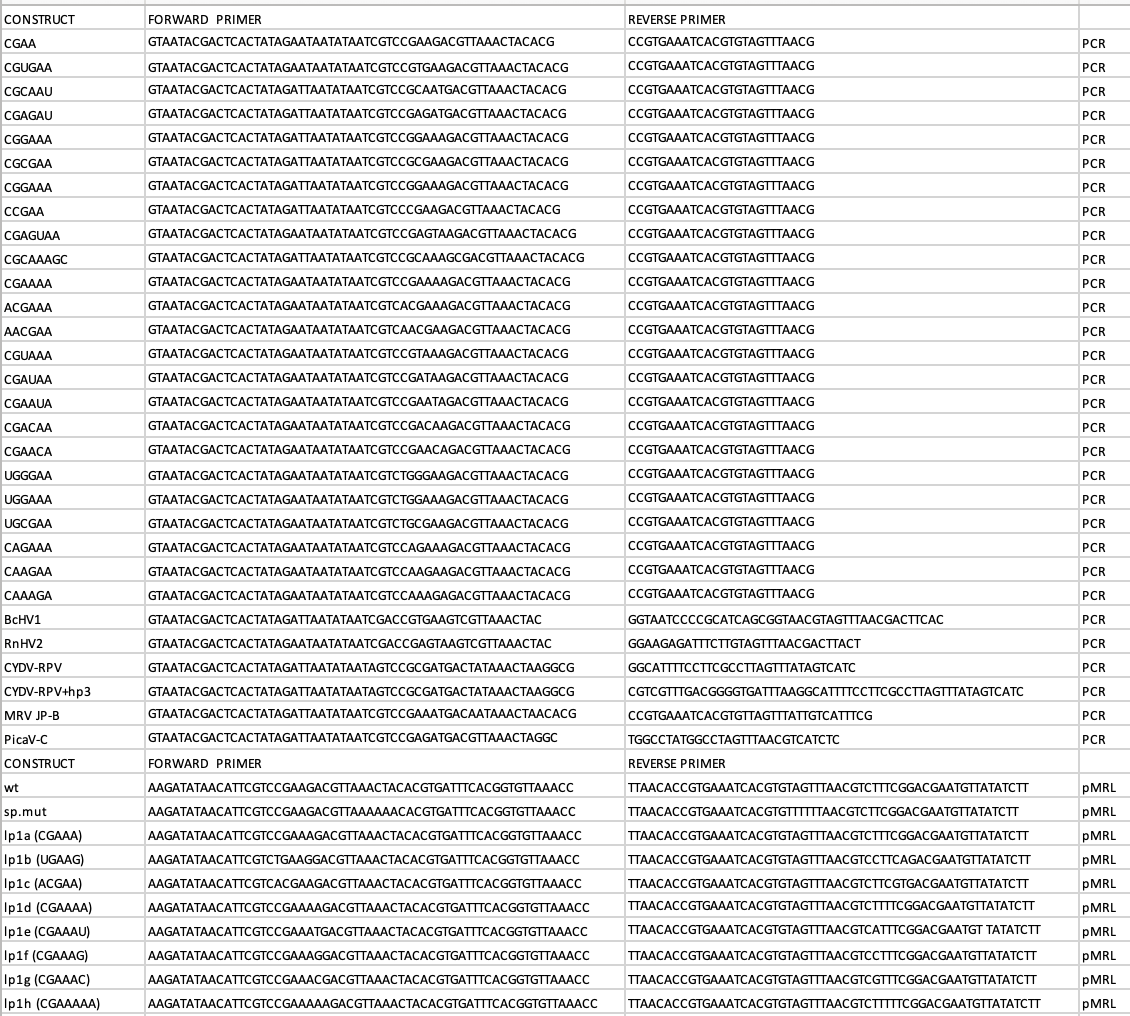


Table S1. Oligonucleotides used for PCR and cloning.
